# Lower Intraoperative and Immediate Postoperative Complications in Robotic Versus Conventional Primary Total Hip Arthroplasty: A Retrospective Cohort Analysis of Over 360,000 Patients

**DOI:** 10.7759/cureus.57726

**Published:** 2024-04-06

**Authors:** Vishesh Khanna, Garrett Sohn, Surya Khanna, Munis Ashraf, Mehul M Mittal, Varatharaj Mounsamy, Senthil Sambandam

**Affiliations:** 1 Orthopedics, Morriston Hospital, Swansea, GBR; 2 Orthopedics, UT Southwestern Medical Center, Dallas, USA; 3 Orthopaedics, Jawaharlal Nehru Medical College, Belagavi, IND; 4 Orthopedics, Saveetha Medical College and Hospital, Chennai, IND

**Keywords:** mortality, morbidity, complications, comparative outcomes, conventional hip arthroplasty, robotic hip arthroplasty

## Abstract

Purpose: Although a trend of an improved alignment with robotic total hip arthroplasty (THA) over conventional methods has surfaced from recent series, it is unknown whether these results translate into meaningful enhancements in postoperative outcomes. To address this lack in the literature, we compared the perioperative morbidity and mortality with robotic and conventional THA in a large national cohort of 367,894 patients. We hypothesized that no significant differences would exist in the outcomes between the two groups.

Methods: Records were extracted from 2016-2019 from the National Inpatient Sample (NIS) database Healthcare Cost and Utilization Project which is the largest in-patient database in the United States. From 367,894 THAs, robotics were employed for 7,863 patients. The remaining 360,031 conventional THAs served as controls. The two groups were compared for demographics, admission, and hospital stay details including costs, and mortality and morbidity data including medical and surgical complications. Descriptive statistics were used for demographic data while analytical statistics including t-tests, chi-squared tests, Fischer exact test, and Pearson chi-squared tests were used for perioperative outcomes. Statistical significance was set at p<0.005.

Results: Demographic distributions between robotic and conventional THA groups displayed similar age and sex characteristics. Shorter mean lengths of stay (1.87 days) were seen in robotic THA versus conventional THA (2.33 days) while higher costs were noted for the former (mean $68,686.71 vs $66,840.39) (p<0.005). Low overall mortality (0.03% robotic, 0.09% conventional) was seen in both groups (p>0.005). Higher comparative incidences of anemia, acute renal failure, and pneumonia were seen in conventional THA (p<0.005) while no significant differences were noted for other complications including myocardial infarction, pulmonary embolism, deep vein thrombosis, and cardiac arrest (p>0.005). Among others, lower dislocation rates, mechanical complications, periprosthetic joint infection, and periprosthetic fractures were seen with robotic THA (p<0.005). Wound complications and superficial infection rates did not differ between the two groups (p>0.005).

Conclusions: Evidence has emerged from our results to support more routine adaptation of the robotic option of performing a THA. These can be based on lower local, systemic, and mechanical complications as demonstrated by the present study. Further evaluation of these results in follow-up would help establish the foothold of robotic surgery in total hip replacement in the modern context.

## Introduction

The implementation of technology in orthopedic surgery has become increasingly evident in the last two decades. This is best illustrated by the amalgamation of navigated and robotic systems employed in total hip arthroplasty (THA). Their use in surgery varies from autonomous function to a more restrictive “guiding” role of the surgeon [1]. Recent studies have reported that the use of robotics in THA has yielded 20-30% improvement in overall implant positioning, including the acetabular cup position and the femoral offset [2]. However, these technical benefits have not necessarily translated to improvements in patient-reported outcomes. Long-term (14-year) follow-up data employing seven different patient-reported outcome measures has demonstrated only minor improvements among two of the seven scores [3]. No significant differences in outcomes have also been reported in a systematic review and meta-analysis involving 14 studies looking at a pool of 1342 patients between conventional, semi-active, or active robotic THA [4]. Additionally, the latter has also been associated with increased costs, operative times (by up to 25 minutes), risks of heterotopic ossification, instability, higher blood transfusion and readmission rates, and the need for revision surgery [5-7].

The more recent analysis demonstrates contrasting evidence from 1,537 patients wherein robotic THA has been seen as favored for lower transfusion rates and length of hospital stay (LoS); furthermore, the authors found a reduction in mean operative time in the robotic sample coinciding with a rise in surgical experience (from 107 2014 to 83 minutes in 2018; p<0.01) [8]. There is a paucity of high-volume data that could address these conflicts of research in determining the safety profile in terms of comparative morbidity and mortality in conventional and robotic THA. Moreover, most of the published studies on outcomes of robotic arthroplasty carry “sponsorship bias,” with over 90% of these having financial conflicts of interest. Unsurprisingly, conflicted studies have been shown to reveal more favorable results with robotic THA when compared with non-conflicted ones [9]. In the era of evidence-based medicine driving patient and surgeon decision-making, the lack of high-volume objective data on robotic THA highlights the need for unbiased evidence on this matter.

In this study, we attempted to compare and contrast the perioperative morbidity and mortality in robotic and conventional THA in an unbiased manner from a large national cohort of over 360,000 patients. In light of conflicting literature findings, we hypothesized that no significant differences would exist in the outcomes between the two groups.

## Materials and methods

Study design

The National Inpatient Sample (NIS) database Healthcare Cost and Utilization Project (HCUP) holds the largest pool of national data in the United States from a fifth of American hospitals for annual admissions, resource utilization, quality, and outcomes for arthroplasties [10]. This database utilizes the International Classification of Diseases, 10th revision codes (ICD-10-CM/PCS) and the present study retrospectively reviewed records from over a four-year period (2016-2019) from the National Inpatient Sample (NIS).

Index event, study population, and outcome

Patients undergoing conventional and robotic THA were identified using the ICD-10 procedural codes and included for analysis. The index event was defined as the day of THA surgery. Revision THAs and complex primary THAs, encompassing surgeries complicated by hip deformities, bone loss, hip joint trauma, complex anatomical variations, and muscular issues, were excluded from the study. The prevalence of robotic THAs among the 367,894 THAs that met the inclusion criteria was 2.1% (7,863 hips). With this data, two cohorts were constructed: robotic THA (n=7,863) and conventional THA (n=359,993). Demographic features, including age at surgery, sex, race, ethnicity, and BMI, were similar between both cohorts; therefore, balancing techniques such as propensity score matching were intentionally omitted to avoid confounding the analysis. The follow-up duration encompassed all inpatient complications from the index event to discharge.

Data collection and analysis

Variables analyzed include demographics (age, sex), type of admission (elective vs non-elective), LoS, costs, and mortality rates. Medical and surgical perioperative complications (renal failure, myocardial infarction, blood loss, pneumonia, thromboembolism, fractures, wound complications, and infections) were also incorporated into the data analysis.

The statistical software Statistical Package for Social Sciences (SPSS), version 27.0 (IBM Corp., Armonk, NY) was used for descriptive and analytical statistics for assessing associations between perioperative factors in conventional and robotic THAs. For age, LoS, and costs, T-tests were used to determine significant differences between the two groups. For binomial variables, the chi-squared tests were used. For incidence values <5, the Fischer exact test was employed whereas Pearson chi-squared tests were utilized for those with values ≥5. Statistical significance was set at p<0.005.

## Results

From a total of 367,894 patients undergoing THAs, 7,863 hips employed the use of a robot. The mean ages of the robotic and conventional THA groups were respectively 64.7 (SD: 11.2) and 65.9 years (SD: 11.4) (p=0.045). The distribution of sex was strikingly similar in the conventional and robotic THA groups with a slight female preponderance of 55% (p=0.101). Among conventional THAs, the incidence of emergency procedures was 8.8% which compared to 3.7% of robotic THAs (Table 1).

**Table 1:** Demographic data in robotic vs conventional THA THA: total hip arthroplasty

Variable	Robotic THA (n=7,863)	Conventional THA (n=359,993)	p
Age at admission (Years)	64.7	65.9	0.045
Sex (Women)	4,326/ 7,863 (55.0%)	201,416/ 359,993 (55.9%)	0.101
Sex (Men)	3,537/ 7,863 (45.0%)	158,577/ 359,993 (44.1%)	
Non-elective	281/ 7,863 (3.7%)	31,553/ 359,529 (8.8%)	<0.001
Elective	7,577/ 7,863 (96.3%)	327,976/ 359,529 (91.2%)	<0.001

The robotic group had shorter mean lengths of stay (1.87 days) versus those with conventional THA (2.33 days, p<0.001). Average costs to health care with conventional hip replacements (mean $66,840.39) were significantly lower (p<0.001) when compared with robotic THA (mean $68,686.71) (Table 2). 

**Table 2:** Length of stay and cost of care in robotic vs conventional THA Tha: total hip arthroplasty

Variable	Robotic THA (n=7,863)	Conventional THA (n=360,031)	p
Mean length of stay (Days)	1.87	2.33	<0.001
Total charges ($)	$68,686.71	$66,848.60	<0.001

Among 367,756 conventional THA patients, an overall mortality rate of 0.09% was observed (Table 3). A comparable mortality of 0.03% was seen for robotic THA patients (Pearson chi-square p=0.053; Fisher’s exact test p=0.054) (Table 3).

**Table 3:** Mortality in robotic vs conventional THA *** HCUP user agreement does not allow publishing values between 1-10 THA: total hip arthroplasty, HCUP: Healthcare Cost and Utilization Project

Variable	Robotic THA (n=7,863)	Conventional THA (n=360,031)	p
Died during hospitalization	*** (0.03%)	330 (0.09%)	0.054

The incidence of postoperative anemia was significantly higher in conventional THA vs their robotic counterparts (19.6% vs 16.1%). In addition, acute renal failure (2.5% vs 1.7%) and pneumonia (0.3% vs 0.1%) were also significantly higher in the conventional THA cohort. There were no significant differences between robotic and conventional THA in rates of myocardial infarction (p=1.000), pulmonary embolism (p=0.494), deep vein thrombosis (p=0.079), and cardiac arrest (p=0.369) (Table 4).

**Table 4:** Systemic complications among robotic vs conventional THA ** Could not be calculated since the value was 0 for robotic patients *** HCUP user agreement does not allow publishing values between 1-10 THA: total hip arthroplasty, HCUP: Healthcare Cost and Utilization Project

Complication	Robotic THA (n=7,863)	Conventional THA (n=360,031)	Odds ratio	(95% CI)	P
Acute renal failure	131 (1.7%)	8,999 (2.5%)	0.661	0.555/ 0.786	<0.001
Myocardial infarction	*** (0.03%)	139 (0.04%)	0.998	0.315/ 3.102	1.000
Cardiac arrest and ventricular fibrillation	0	37 (0.01%)	**	**	0.369
Anemia	1,263 (16.1%)	70,708/ 360,031 (19.6%)	0.783	0.737/ 0.832	<0.001
Pneumonia	*** (0.1%)	964 (0.3%)	0.427	0.221/ 0.823	0.009
Pulmonary embolism	*** (0.1%)	467 (0.1%)	0.784	0.390/ 1.578	0.494
Deep vein thrombosis	*** (0.1%)	556 (0.2%)	0.494	0.221/ 1.104	0.079

The robotic THA group exhibited significantly lower rates of postoperative dislocations (0.1% vs 1.4%) and periprosthetic joint infections (0.03% vs 1%), inclusive of infections and inflammatory reactions related to the hip prosthesis. Additionally, lower rates of mechanical complications, which include implant loosening, malalignment, and soft tissue irritation, were seen in the robotic THA group (0.1% vs 0.8%). Furthermore, the rate of postoperative periprosthetic fractures was also three times lower in robotic THAs (0.4% vs 1.2%). There were no significant differences in rates of wound dehiscence (p=0.160) and superficial (p=0.332) and deep (p=0.418) incisional surgical site infections (Table 5).

**Table 5:** Local complications among robotic vs conventional THA ** Could not be calculated since the value was 0 for robotic patients *** HCUP user agreement does not allow publishing values between 1-10 THA: total hip arthroplasty, HCUP: Healthcare Cost and Utilization Project, SSI: surgical site infection

Complication	Robotic THA (n=7,863)	Conventional THA (n=360,031)	Odds Ratio	(95% CI)	P
Periprosthetic fracture	29 (0.4%)	4,396/ 360,031 (1.2%)	0.299	0.208/ 0.432	<0.001
Periprosthetic dislocation	*** (0.1%)	5,145/ 367,511 (1.4%)	0.053	0.024/ 0.117	<0.001
Periprosthetic mechanical complications	*** (0.1%)	2,851/ 360,031 (0.8%)	0.080	0.033/ 0.192	<0.001
Wound dehiscence	*** (0.04%)	304/ 360,031 (0.08%)	0.452	0.145/ 1.408	0.160
Superficial SSI	0	43/ 360,031 (0.01%)	**	**	0.332
Deep SSI	0	30/ 360,031 (0.01%)	**	**	0.418
Periprosthetic joint infection	*** (0.03%)	3,828/ 360,031 (1%)	0.036	0.011/ 0.110	<0.001

## Discussion

To the best of our knowledge, this study represents the largest analysis of postoperative outcomes of conventional versus robotic THAs. Our findings depict results from a national large data set over a four-year period (2016-2019). These are aimed at bringing out differences between robotic and conventional THA outcomes in the postoperative period leading up to discharge from the hospital.

The contention of whether including the use of robotics in hip arthroplasty actually imparts any proven benefit has halted their widespread application to arthroplasty centers across the contemporary world. Although there has been a 30-fold increase in the incidence of technology-assisted THA from 0.1% in 2005 to 3% in 2015 (based on NIS data), the curve seems to have plateaued over the last decade [11]. The present paper for robotic THA cites a 2.1% incidence among all THAs performed from 2016 to 2019. The objective illustration of postoperative outcomes by our results has allowed the identification of clear advantages and drawbacks of this technology in an unconflicted manner. To our knowledge, this is the largest study done so far looking at post-operative outcomes between robotic and conventional hip replacement.

The hypothesis that there would be no significant differences between the two groups proved incorrect. The results from our analysis revealed a slightly younger population receiving robotic THA. Perhaps this reflects an inverse relation between acceptance of this technology and age [7, 12-15]. Women have proportionately dominated both groups with higher prevalence in the present paper, and this aligns with previously published series [7,12-14].

Previous literature has demonstrated a shorter hospital length of stay associated with robotic THA and the results of our study coincide with this finding [7, 16-18]. A clear explanation for this phenomenon has not been described in the published literature. However, it is possible that institutions supporting robotic surgery are likely to have more standardized, “fast-track” protocols enabling earlier discharge times [17]. Higher costs were seen for robotic THAs by nearly $2,000 per patient in our results. A similar average cost was described in another recent study by Kirchner et al. based on NIS data from 2010-2014 among 758 matched robotic and conventional THA patients [18]. Interestingly, one study by Remily et al. demonstrated a reduction in overall costs with robotic THAs [7]. Many reasons were cited to account for this, including shortened LoS and reduced longer-term cumulative costs, which was illustrated by a Markov model analysis by Maldonado et al. [19]. The authors found robotic THA more cost-effective from the payer’s perspective in both Medicare and private paying scenarios by cumulative cost differences at five years of $945 and $1,810, respectively [19]. Clement et al. evaluated incremental cost-effectiveness ratios and a lifetime horizon to report lower (£452) unadjusted cost per quality-adjusted life year with robotic THA [20]. Higher mean surgical times have been associated with robotic THAs, likely a result of surgeon learning curves in using robotic systems. Several researchers have suggested that surgical times could be sequentially decreased as proficiency increases. Simcox et al. showed that there was a reduction in the operating time from 36 minutes in 2010 to 15 minutes in 2018, likely due to improved technology and adaptation around the surgical method with the robot [17].

In our study, improvement in instrumentation techniques and surgical expertise could probably explain a relatively lower incidence of anemia, acute renal failure, and pneumonia among robotic THAs. Blood loss was more consistently reported higher with robotic THA from studies in the past and a shift in this regard does represent a positive change [7,17-18]. Among mechanical complications, lower rates of perioperative dislocations and fractures with robotic THA could also likely be attributed to the more optimal positioning of components in our series. This principal advantage with the use of robots and has been described extensively [1-3,6,21]. 

Two previous studies have looked at perioperative comparative outcomes between robotic and conventional THA using similar large national databases [17,18]. Kirchner et al. have evaluated matched 758 patients from both conventional and robotic THA cohorts, and they report a slightly higher risk of minor complications, higher hospital costs, and shorter LoS among the robotic group [18]. Although similar to our present study, the above studies also employed the NIS database, their use of relatively smaller datasets that are now nearly a decade old limits the wider application of their inferences. This could also account for their reports of higher complication rates associated with robotic THA, which contrasts our present results.

In another study, Simcox et al. examined a wider population of 238,755 THAs over 8 years from the American College of Surgeons National Surgical Quality Improvement Program database [17]. They reported lower transfusion rates and shorter hospital stays in the robotic THA group, which our findings concur with. These results illustrate the importance of large-volume data studies, and how these analyses can derive more conclusive and agreeable inferences even from a different set of patients. Some limitations of the study by Simcox et al. include the absence of cost assessment, a long study period (2010-2018) with a relatively lower sample of 238,755 patients, and heterogeneous data including all forms of technology-assisted THA [17]. Understandably, surgical practices and standards of care would have evolved more over the 8-year period and this could potentially have skewed the outcomes [17]. Our study addresses these shortcomings by comprehensively reporting on nearly 370,000 patients in consecutive years (2016-2019) from among a nationwide data analysis. For reference, highlights from other studies comparing complications after robotic and conventional THA are included in Table 6.

**Table 6:** Complications after robotic and conventional THA within published literature THA: total hip arthroplasty: LoS: length of hospital stay

Authors	Year	Robotic THA (n)	Conventional THA (n)	Favor conventional THA	Favor robotic THA
Singh et al. [16]	2016-2020	135	929	Increased mean surgical time (119.6 vs 95.35 mins)	Longer LoS (mean 2.22 vs 1.91 d)
Remily et al. [7]	2010-2018	4,630	4,630	Higher readmission rates (7.8% vs 6.6%), higher blood transfusion rates (4.4% vs 3.2%)	Longer LoS (3.7 vs 3.4 d), higher 90-d costs ($16,633 vs 15,454), and 1-year costs ($24,050 vs $22,011)
Chen et al. [22]	2017	522	994	Longer surgical time, risk of heterotopic ossification	Higher intraoperative complication rates
Han et al. [21]	1998-2018 (MA)	462	615	Longer surgical time, higher postoperative complications (dislocation and revision)	Higher intraoperative complications
Simcox et al. [17]	2010-18	3,149	235,606	Longer mean surgical time (101 vs 91.9 mins), higher readmission rates (3.8% vs 2.4%)	Higher blood transfusion rates (7.8% vs 5.7%), longer LoS (2.5 vs 2 days)
Kirchner et al. [18]	2010-2014	758	758	Higher risk of “minor complications,” higher costs	Longer LoS (2.82 vs 2.69 days)
Present Study	2016-19	7863	360,031		Higher risk of complications

The authors are conscious of the limitations of this research including those from the employment of national data from the immediate postoperative phase. With the reputation of being largely accurate, there could possibly have been a few missing patients without complete information. The unavailability of certain details such as surgical time also does not provide a comparative evaluation in this regard. It has, however, been established through meta-analyses that surgical times are typically longer with robotic THA. Essentially a short-term study, the paper also cannot comment on whether the differences noted would persevere in the mid-long-term follow-ups. The sheer volume of data of nearly 370,000 patients also limits the comparative assessment of radiological parameters such as implant positioning between the two groups. Moreover, the safe zones to determine cup positioning have been challenged in their accuracy and have been varyingly modified and described in the literature [23]. Finally, our analysis was unable to restrict itself solely to patients with specific surgical pathologies or to certain surgical approaches; nonetheless, considering the scale of our study, we believe these limitations are largely mitigated. Current prospective studies underway may alleviate the majority of concerns associated with our study, given they are less prone to errors in documentation, discrepancies in indication, and the presence of correlation fallacies commonly found in retrospective analyses.

## Conclusions

In summary, the present era reveals mounting evidence favoring the more frequent utilization of robotic total hip arthroplasty (THA). Our findings demonstrate reduced complication and morbidity rates associated with this technology. Further research is warranted to reduce the limitations inherent to retrospective studies such as ours and to better elucidate in which indications robotic THA may be more favorable versus conventional THA.
